# Atomic Origin of Interface‐Dependent Oxygen Migration by Electrochemical Gating at the LaAlO_3_–SrTiO_3_ Heterointerface

**DOI:** 10.1002/advs.202000729

**Published:** 2020-06-28

**Authors:** Dongsheng Song, Deqing Xue, Shengwei Zeng, Changjian Li, Thirumalai Venkatesan, Ariando Ariando, Stephen J. Pennycook

**Affiliations:** ^1^ Department of Materials Science and Engineering National University of Singapore Singapore 117575 Singapore; ^2^ NUSNNI‐Nanocore National University of Singapore Singapore 117411 Singapore; ^3^ Department of Physics National University of Singapore Singapore 117542 Singapore

**Keywords:** electrochemical gating, ionic liquid gating, LaAlO_3_/SrTiO_3_, oxygen migration

## Abstract

Electrical control of material properties based on ionic liquids (IL) has seen great development and emerging applications in the field of functional oxides, mainly understood by the electrostatic and electrochemical gating mechanisms. Compared to the fast, flexible, and reproducible electrostatic gating, electrochemical gating is less controllable owing to the complex behaviors of ion migration. Here, the interface‐dependent oxygen migration by electrochemical gating is resolved at the atomic scale in the LaAlO_3_–SrTiO_3_ system through ex situ IL gating experiments and on‐site atomic‐resolution characterization. The difference between interface structures leads to the controllable electrochemical oxygen migration by filling oxygen vacancies. The findings not only provide an atomic‐scale insight into the origin of interface‐dependent electrochemical gating but also demonstrate an effective way of engineering interface structure to control the electrochemical gating.

## Graphical abstract

The interface‐dependent oxygen migration by electrochemical gating is resolved at the atomic scale in the LaAlO_3_–SrTiO_3_ system through ex situ ionic liquid gating experiments and on‐site atomic‐resolution characterization. The difference between interface structures leads to the controllable electrochemical oxygen migration by filling oxygen vacancies.

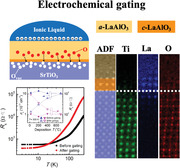

## 1 Introduction

The electric field effect with ionic liquids (ILs) as dielectrics has been widely used to control the electronic, magnetic, and optical properties of functional oxides.^[^ ^1^ ^]^ This effect was previously thought to be electrostatic doping^[^ ^2^ ^]^ by the electric double‐layer transistor (EDLT)^[^ ^3^, ^4^, ^5^, ^6^, ^7^ ^]^ and has already been demonstrated to induce or control superconductivity,^[^ ^8^, ^9^, ^10^, ^11^, ^12^ ^]^ ferromagnetism,^[^ ^13^, ^14^, ^15^, ^16^ ^]^ and the metal–insulator transition (MIT).^[^ ^16^, ^17^ ^]^ Later, it turns out that the purely electrostatic mechanism is not solely existing during IL gating, which is understood as electric field‐induced oxygen migration,^[^ ^18^ ^]^ termed the electrochemical mechanism. The competition between electrostatic and electrochemical mechanisms has been reported in many binary^[^ ^17^, ^18^, ^19^, ^20^, ^21^, ^22^, ^23^, ^24^ ^]^ and complex oxides.^[^ ^6^, ^11^, ^14^, ^25^, ^26^, ^27^, ^28^, ^29^ ^]^ The electrochemical mechanism can be greatly suppressed through the insertion of inert layers between the IL and the materials, such as ultrathin boron nitride^[^ ^30^ ^]^ or graphene.^[^ ^31^ ^]^ It is also applicable to a 2D electron gas (2DEG) system, such as Al_2_O_3_–SrTiO_3_ (STO),^[^ ^32^ ^]^ crystalline LaAlO_3_ (*c*‐LAO)–STO,^[^ ^33^, ^34^ ^]^ for which the capping layers (e.g., Al_2_O_3_, *c*‐LAO) simultaneously serve as the protective layers to guarantee the purely electrostatic gating of the buried oxide interface. However, the transport properties of the 2DEG can be electrochemically gated by an IL in the buried interface for amorphous LAO (*a*‐LAO)^[^ ^27^ ^]^ and crystalline CaZrO_3_^[^ ^35^ ^]^ on STO. Similarly, buried VO_2_ can still be reversibly gated through electrochemical oxygen migration, even with an inserted TiO_2_ layer up to 10 nm.^[^ ^22^ ^]^ Therefore, electrochemical oxygen migration in differently gated material systems exhibit different behaviors, making electrochemical gating less flexible and controllable.

Here, we demonstrate that modifying the interface structure is an effective and practical way to make the electrochemical oxygen migration controllable. Taking the archetypical 2DEG at the LAO/STO interface, ex situ IL gating experiments and on‐site atomic‐scale characterization by aberration‐corrected scanning transmission electron microscopy (STEM) evidence the correlation of interface structure and electrochemical oxygen migration. It is shown that the electrochemical oxygen migration process of filling oxygen vacancies in STO can be controlled during the IL gating through engineering the atomic structure of the interfacial LAO overlayer.

## 2 Results and Discussion

**Figure 1:**
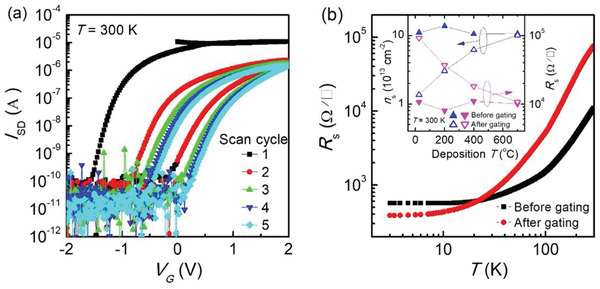
a) Source–drain current *I*_SD_ as a function of gate voltage *V*_G_ for *a*‐LAO/STO grown at 25 °C. The scanning for each cycle starts from 0 to +2 to −2 V and stops at 0 V. b) Sheet resistance *R*_s_ as a function of temperature *T* for *a*‐LAO/STO grown at 25 °C, before and after gating. Inset of (b) shows the sheet carrier density *n*_s_ and *R*_s_ at 300 K for LAO/STO grown at different temperatures of 25, 200, 400, and 700 °C, before and after gating.

The LAO thin films, with a thickness of ≈5 nm, are grown on (001) orientated STO substrates at different temperatures of 25 (room temperature, RT), 200, 400, and 700 °C using a pulsed laser deposition (PLD) system. The oxygen partial pressure *P*_O2_ is 2 × 10^−4 ^Torr to produce oxygen‐deficient LAO/STO interfaces during the deposition and no post oxygen annealing is performed to recover the oxygen stoichiometry at the interface. The room‐temperature carrier density is ≈1 × 10^14^ cm^−2^ for the samples deposited at different temperatures as shown in the inset of **Figure** **1**, which is almost one order of magnitude higher than that of the oxygen‐annealed crystalline LAO/STO, making the oxygen vacancies as the predominant origin of the 2DEG.^[^ ^36^, ^37^ ^]^ These samples are divided into two groups: one is in the as‐grown state and the other is processed with IL gating to push the oxygen ions from the LAO overlayers into the STO to partially fill the oxygen vacancies. Figure 1a shows an example of IL gating for the LAO/STO interface grown at 25 °C, by scanning source–drain current *I*_SD_ as a function of gate voltage *V*_G_ between −2 and 2 V. The irreversible and hysteretic behavior in the *I*_SD_–*V*_G_ curve indicates that oxygen electromigration occurs during IL gating and oxygen vacancies are filled at negative *V*_G_.^[^ ^27^ ^]^ In addition, the *I*_SD_–*V*_G_ curves for the LAO/STO grown at *T*_d_ = 200, 400, and 700 °C are shown in Figure S1 in the Supporting Information. These data coupled with the one for *T*_d_ = 25 °C show that the samples deposited at low temperature (*T*_d_ = 25 and 200 °C) exhibit large hysteresis and irreversibility while samples deposited at high temperature (*T*_d_ = 400 and 700 °C) exhibit small hysteresis and irreversibility, especially for the first scan cycle. Moreover, *I*_SD_–*V*_G_ curve for oxygen‐annealed crystalline LAO/STO (*T*_d_ = 700 °C) is also measured and shows a reversible and nonhysteretic metal–insulator transition in Figure S2 in the Supporting Information, indicating the pure electrostatic charging and depletion.^[^ ^33^ ^]^ In general, with increasing deposition temperatures, the LAO layers change from amorphous to crystalline, indicating that the oxygen filling effect is obvious in amorphous samples. Figure 1b shows the sheet resistance *R*_s_ versus temperature *T* curves for the sample grown at 25 °C before and after gating. One can see that the metallic nature is preserved after gating with an increase in *R*_s_ at room temperature. Note that the resistance before gating is higher than that after gating at the low‐temperature range. This might be due to the presence of oxygen vacancies in the as‐grown sample, which could act as scattering centers and cause the relatively high resistance at low temperatures. After the filling of oxygen vacancies through gating, the scattering centers are reduced and the decrease of low‐temperature resistance is observed, similar to previous reports.^[^ ^36^, ^40^ ^]^ IL gating for LAO/STO interfaces at all four growth temperatures is performed and the room‐temperature sheet carrier density *n*_s_ and *R*_s_ before and after gating are shown in the inset of Figure 1b. The *R*_s_ increases and *n*_s_ decreases by one order of magnitude after gating for the sample grown at 25 °C. The *R*_s_ and *n*_s_ show less change after gating as the grown temperature increases, and at 700 °C, the change is negligible. In addition, the source–drain current *I*_SD_ as a function of time is also shown in Figure S3 in the Supporting Information with the negative gate voltage *V*_G_ applied for samples at different *T*_d_. At high *T*_d_, the *I*_SD_ almost recovers to its initial value as the *V*_G_ is off, indicating a negligible filling effect for the crystalline sample, consistent with the changes of scanning of *V*_G_. The evolution of the change of *R*_s_ and *n*_s_ after gating, induced by the filling of oxygen vacancies in STO, is affected by the growth temperature, indicating the strong dependence of the electrochemical mechanism on the underlying interface structure.

**Figure 2:**
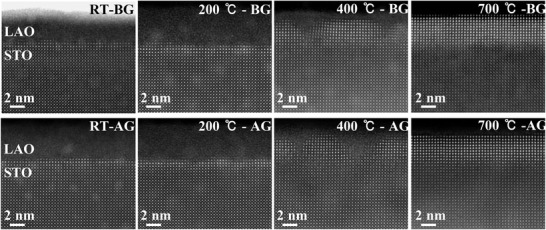
Atomic resolution HAADF–STEM images of LAO/STO interfaces grown at different temperatures before and after the IL gating, taken along the [100] zone axis. The surfaces of LAO thin films are protected with Au or carbon thin films before the preparation of TEM samples.

To clarify the correlation between the interface structure and oxygen migration, STEM measurements are conducted on the samples both before and after scanning as a function of gate voltage for five cycles, for which the *R*_s_ and *n*_s_ have already been changed through the electromigration of oxygen ions in Figure 1b. **Figure** **2** shows the atomic resolution high angle annular dark field STEM (HAADF–STEM) images of the LAO/STO interfaces. The interfaces show almost no change of morphology and atomic structure before and after gating, indicating that the LAO overlayers not only supply the oxygen ions to fill the oxygen vacancies but also act as a protective layer to effectively prevent the chemical reaction between the IL and the buried interface. The LAO overlayers evolve from amorphous at RT to a fully crystalline state at 700 °C with the increasing growth temperature. From RT to 200 °C, the LAO is amorphous but the interface appears to be bright within one‐ or two‐unit cells. For the sample grown at 400 °C, crystalline LAO islands appear to be separated by amorphous LAO. Meanwhile, these islands are still coherent with the STO substrate and the degree of crystallinity is gradually decreased from the interface to the surface. The surface layer within ≈1 nm is still amorphous over the whole thin film.

The LAO overlayers serve as the source of oxygen ions which are injected into the STO layers under the electric field. The formation energy of oxygen vacancies in *a*‐LAO is considerably lower than that in crystalline LAO. Therefore, the nontunability of carrier density by electrochemical IL gating for the 700 °C sample can be attributed to the full crystallinity of the LAO overlayer. For other samples grown at lower temperatures, the *a*‐LAO layers provide the oxygen source and the consequent oxygen migration to fill the oxygen vacancies in STO under the electric field. However, the fraction of the filled oxygen vacancies in STO shows a big difference according to the inset of Figure 1b. The interface between the *a*‐LAO and STO, which is the only route for transporting oxygen into STO, should account for this discrepancy.

**Figure 3:**
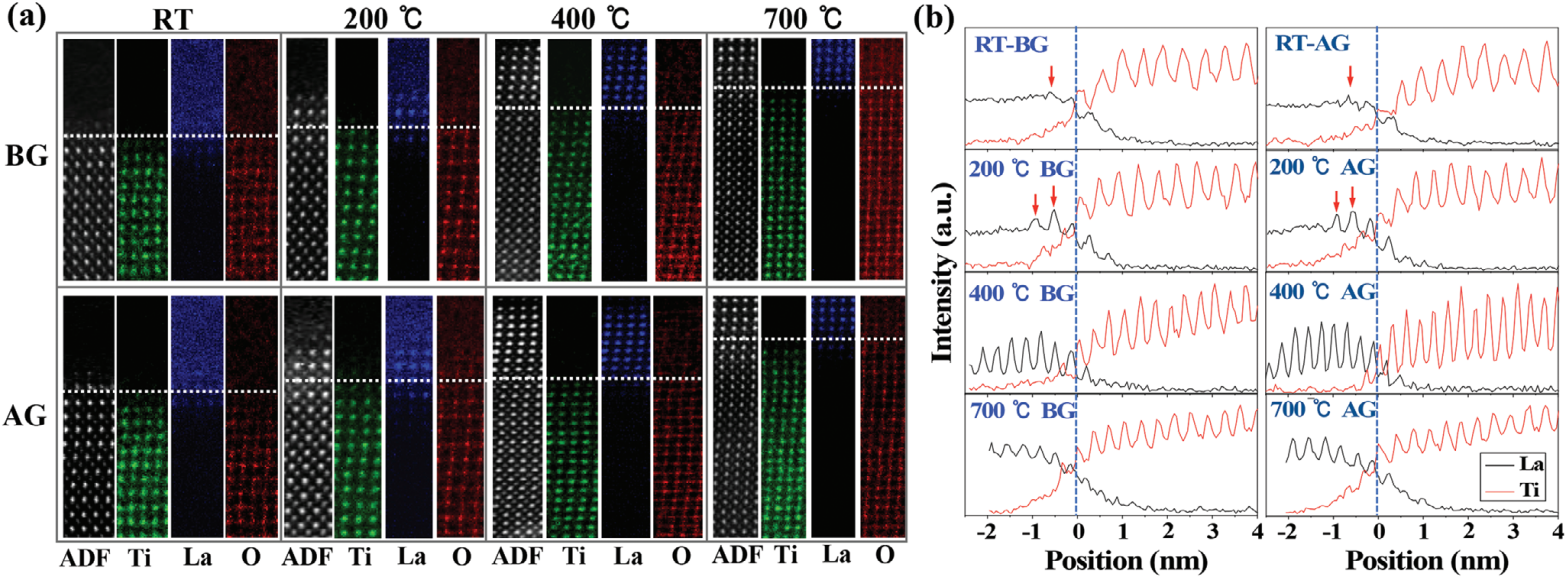
Chemical composition and atomic structures across the LAO/STO interface grown at different temperatures before and after IL gating. a) Atomic resolution elemental mapping of La (blue), Ti (green) and O (red), and the simultaneously acquired ADF images. b) The integrated intensity profiles of La and Ti from (a). The interface is indicated by dotted lines.

The elemental mappings of La, Ti, and O across the interface by atomic resolution electron energy‐loss spectroscopy (EELS) are displayed in **Figure** **3**a. The integrated line intensity profiles of Ti and La are also plotted together in Figure 3b for comparison. One can see that the chemical compositions show no obvious difference before and after IL gating, indicating that the buried interfaces are well protected. The differences between these samples are mainly reflected in the interface structures, showing an additional LAO layer with various thickness and degree of crystallinity. For the RT grown sample, the ordered LAO is only one monolayer in thickness as indicated by red arrows in Figure 3b. The weak contrast of the La lattice in Figure 3a indicates that the monolayer LAO is not fully crystalized. For the sample grown at 200 °C, the thickness of the crystalline LAO layer is increased to 2–3 unit‐cells with an increased degree of crystallinity, as the contrast of La elemental mapping becomes much more obvious in Figure 3a. The 400 °C grown sample exhibits a much wider region of crystalized LAO layer. Also, the decreased intensity of the La profile approaching the surface indicates the decreasing degree of crystallinity for the 400 °C grown sample.

**Figure 4:**
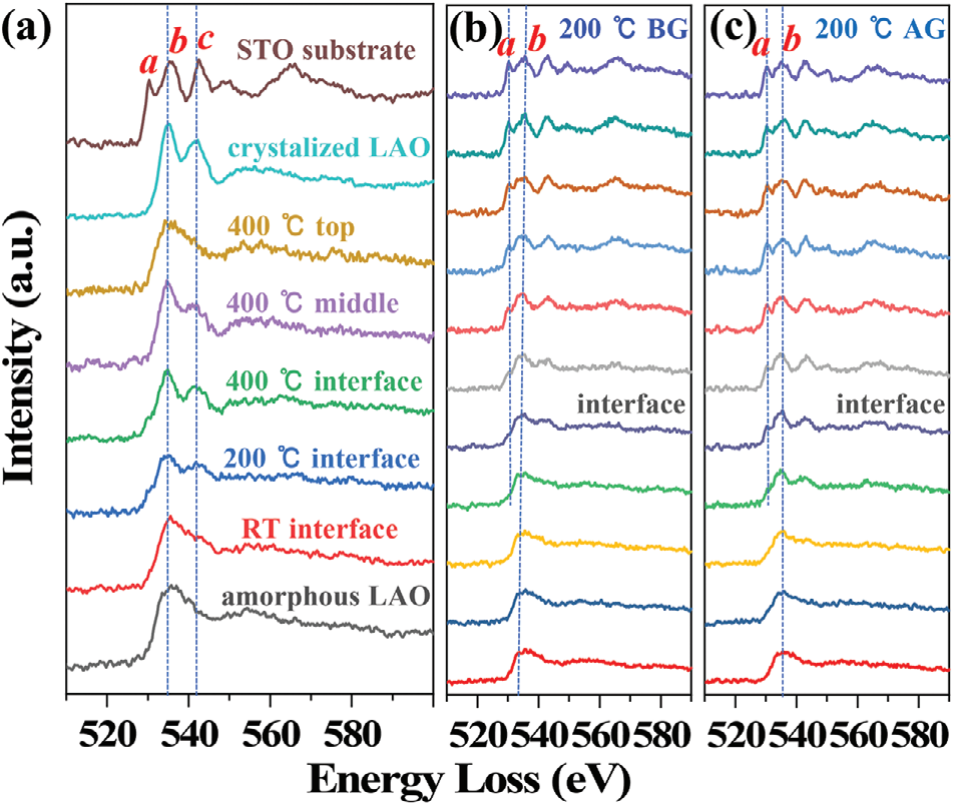
Revealing the interface states of LAO/STO by the fine structures of the O *K* edge. a) EELS O *K* edge extracted from different regions of LAO/STO grown at different temperatures before IL gating. The crystalline and amorphous LAO samples are grown at 700 °C and RT, respectively. The peaks of O *K* edge are indicated with *a*, *b*, and *c* taking the reference of STO. b,c) The EELS O *K* edge before and after IL gating across the LAO/STO interface, respectively, for the sample grown at 200 °C. The positions of peak *a* and *b* are indicated by dotted lines.

The difference of the interface structures is also reflected in the EELS O *K* edge as shown in **Figure** **4**a, which are extracted from the different regions of LAO layers grown at different temperatures. The peaks *a*, *b*, and *c* are indicated in the EELS O *K* edge in Figure 4a, respectively. Peak *a* corresponds to the electron transition from O 2p to Ti 3d orbital in STO layers while being absent in LAO layers. The intensity of peak *a* and the energy shift between peak *a* and *b* can be used to qualitatively judge the concentration of oxygen vacancy at the interface. Peak *c* is related to the atom coordinate environment surrounding the oxygen and therefore can be used to judge the degree of crystallinity in LAO layers. The disappearance of peak *c* corresponds to the amorphous state of LAO layers. By comparing the EELS respectively from fully crystallized and amorphous LAO regions, it can be seen that the sample grown at RT exhibits a weak peak *c* at the LAO side of the interface. The peak *c* becomes more obvious for the sample grown at 200 °C, indicating an increased degree of crystalline. For the sample grown at 400 °C, it is fully crystallized at the interface with very sharp peaks *b* and *c*. Approaching the surface, the degree of crystallinity is seen to gradually decrease with the decreased intensity of peak *c*. At the surface, the LAO layer returns to the amorphous state.

Referring to the tunability of carrier density by IL gating for the samples grown at low temperature in Figure 1, the interface structures are responsible for it. The oxygen ions firstly can move toward the interface through the amorphous LAO layers with a lower energy barrier under the electric field.^[^ ^38^, ^39^ ^]^ Next, the interfacial LAO structures build another barrier layer. The interface with a lower degree of crystallinity holds a loose lattice structure, in which the oxygen ions might readily diffuse under the electric field as revealed in previous reports by first‐principles molecular dynamics simulations.^[^ ^39^ ^]^ The increased degree of crystalline can significantly affect the amount of filled oxygen vacancies by changing the carrier density from Δ*n*_s_/*n*_s_ ≈ 0.85 (grown at RT) to Δ*n*_s_/*n*_s_ ≈ 0.69 (grown at 200 °C) in Figure 1, respectively, demonstrating the significant role of the interface layer (even with a thickness of one or two unit‐cells) in controlling the transport properties as reported before.^[^ ^40^ ^]^ For the sample grown at 400 °C, the electrochemical oxygen migration is greatly suppressed by the crystallized LAO layers in most areas. While for areas between the crystalline LAO islands, which are a mixture of amorphous and a thin crystalized LAO layers, the oxygen migration can still occur even with suppression of Δ*n*_s_/*n*_s_ ≈ 0.42. For the sample grown at 700 °C, the oxygen migration is significantly blocked (Δ*n*_s_/*n*_s_ ≈ 0.06) by the fully crystallized LAO layer. Therefore, it is the interface structure, even with slight modifications, that determines the controllable electrochemical oxygen migration.

Furthermore, the evidence of filling oxygen vacancies by IL gating is provided to directly certify the mechanism of interface‐dependent electrochemical oxygen migration at the atomic scale. The intensity and energy shift of the O *K* edge peaks can be used to identify the interface oxygen vacancies,^[^ ^41^, ^42^ ^]^ as shown in Figure 4b,c, taking the example of the sample grown at 200 °C (see others in Figure S4 in the Supporting Information). The energy shift between peak *a* and *b* (indicated with dotted lines) is decreased on approaching the interface in the STO side for the sample before gating in comparison to the sample after gating. The smaller energy shift indicates a higher concentration of oxygen vacancies. Meanwhile, the intensity of peak *a* is greatly decreased at the interface in the sample before gating due to the existing of oxygen vacancies in Figure 4b but is mostly recovered in Figure 4c due to the filling of oxygen vacancies after gating. These features definitively demonstrate that the oxygen vacancies are filled through electrochemical oxygen migration.

**Figure 5:**
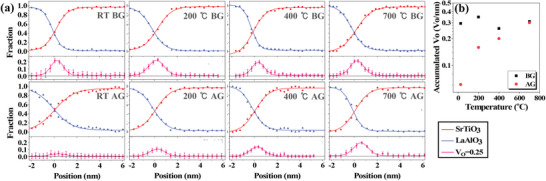
Quantifying the amount of filled oxygen vacancies by electrochemical oxygen migration during IL gating. a) The fitting results of STO and LAO, and the distribution of oxygen vacancies (*V*_O_ = 0.25) across the LAO–STO interfaces. b) The total amount of accumulated oxygen vacancies at the LAO–STO interface before and after IL gating, respectively.

To quantify the amount of the filled oxygen vacancies at the LAO/STO interface before and after IL gating, the fine structures of the EELS O *K* edge are further analyzed. The multiple linear least squares (MLLS) fitting approach^[^ ^43^ ^]^ is applied and four reference spectra of EELS O *K* edges are used. Three of them, the O *K* edge in amorphous LAO layer, fully crystalized LAO layer, and bulk STO, are taken from the experimental EELS results. An additional EELS O *K* edge of oxygen‐deficient SrTiO_2.75_ is taken from reference,^[^ ^44^ ^]^ which implies 0.25 oxygen vacancy per unit cell (*V*_O_ = 0.25). The distributions of oxygen vacancies across the interface are quantitatively determined as shown in **Figure** **5**a. The fractions of oxygen vacancies are almost the same for all the samples before gating, consistent with the macroscopic measurement of *n*_s_ in the inset of Figure 1b. The profiles of oxygen vacancies extend ≈2 nm into the STO substrate with the maximum concentration almost at the interface according to the profiles of LAO and STO. After the IL gating, the fractions of oxygen vacancies are decreased owing to the electrochemical oxygen migration as shown in the lower panel of Figure 5a. After integrating the concentration of oxygen vacancies across the interface for all the measured samples in Figure 5b, the total amount of residual oxygen vacancies increases with increasing growth temperature, indicating the suppression of electrochemical oxygen migration. Regardless of the negligible difference of total accumulated oxygen vacancies at the LAO–STO interface before and after gating at 700 °C in Figure 5b, the shift of the peak position in oxygen vacancy distribution is observed toward STO layer after gating in Figure 5a. It might be caused by the relatively higher degree of diffusion at the EELS measured regions of the gated sample in the experiments, which could be hardly avoided for the sample grown at high temperatures. The concentration of oxygen vacancies for the samples before and after gating shows a similar trend to the carrier density in the inset of Figure 1b. These results not only prove the mechanism of electrochemical oxygen migration during the IL gating at the atomic scale but also demonstrate the strong interface‐dependent electrochemical IL gating.

**Figure 6:**
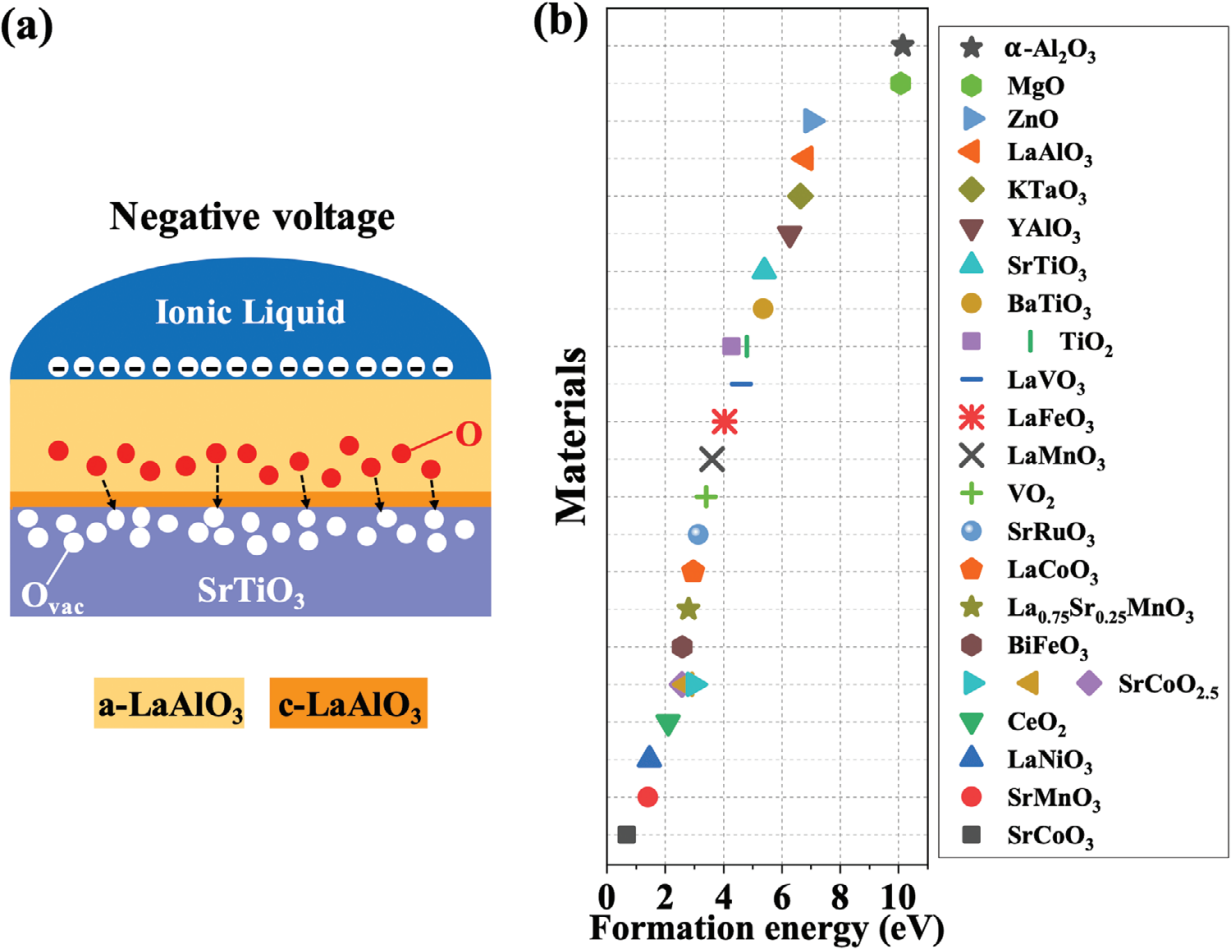
Schematics of electrochemical oxygen migration. a) The migration behavior of oxygen vacancies (ions) with a negative voltage during the IL gating in the case of LAO–STO. b) The formation energy of oxygen vacancies for some typical functional oxides.

To fill the oxygen vacancies by IL gating, a negative voltage is applied as shown in **Figure** **6**a. The movement of oxygen ions is accompanied by the generation of oxygen vacancies in the LAO layer. Meanwhile, the creation of oxygen vacancies can also supply the equivalent sites, allowing the oxygen ions to migrate with a lower energy barrier, which is similar to the case of oxygen ion conductors.^[^ ^45^ ^]^ The migration path, energy barrier (*E*_Odiff_) and the oxygen vacancy formation energy (*E*_Ov_) determine the oxygen ion transport at the interface. The *E*_Odiff_ in amorphous LAO is lower than that in crystalized LAO. The interfacial crystalized LAO layer with a large thickness would increase the migration path length. The smaller *E*_Ov_ could help to easily create the oxygen vacancies and lower the *E*_Odiff_. Therefore, the interface structures, from a thin and poorly crystalline LAO layer to a thick and fully crystalized LAO layer, will lead to different behaviors in the filling of oxygen vacancies. Consequently, the electrochemical oxygen migration process can be controlled through engineering the interfacial LAO structure.

Although the interface‐dependent electrochemical oxygen migration by IL gating is only experimentally demonstrated in the LAO/STO system, it can also be generalized to other materials systems by controlling the thickness, morphology or material type of the interface structure. More importantly, the interface structure can be determined during thin film deposition rather than post processing,^[^ ^30^, ^31^ ^]^ which we have shown can be controlled to an accuracy of a single atomic layer. As the *E*_Ov_ is intrinsic to the material type, we also summarize the *E*_Ov_ for several common oxides in Figure 6b. The lower *E*_Ov_ for VO_2_ (3.40 eV)^[^ ^46^ ^]^ and TiO_2_ (4.27 eV, anatase)^[^ ^47^ ^]^ can explain the reversible electrochemical gating of buried VO_2_ thin films under the TiO_2_ layers.^[^ ^22^ ^]^ However, the high *E*_Ov_ for STO (5.40 eV)^[^ ^48^ ^]^ leads to irreversible electrochemical gating in our LAO/STO systems, as the energy to create oxygen vacancies in crystalline STO is relatively high and the electrochemical gating induced oxygen migration might be suppressed when applying a reversed (positive) gating voltage. Similarly, the electrochemical oxygen migration by IL gating has also been demonstrated in the crystalline LaFeO_3_/STO 2DEG system.^[^ ^25^ ^]^ In comparison with our *c*‐LAO/STO system, which shows negligible oxygen migration during IL gating, the large difference of *E*_Ov_ for LAO (6.80 eV)^[^ ^48^ ^]^ and LaFeO_3_ (4.04 eV)^[^ ^49^ ^]^ should be the underlying reason. Furthermore, some other common oxides, such as LaMnO_3_ (3.6 eV),^[^ ^48^ ^]^ SrCoO_2.5_ (2.57–2.97 eV),^[^ ^50^ ^]^ YAlO_3_ (6.27 eV)^[^ ^49^ ^]^ and *α*‐Al_2_O_3_ (10.14 eV),^[^ ^51^ ^]^ can be explored to improve or suppress the controllable electrochemical gating efficiency in future studies.

Lastly, the effect of the capping layer thickness is discussed on the electrochemical oxygen migration during the gating. The gating experiments have also been conducted on the *a*‐LAO/STO samples (*T*_d_ = 25 °C) with LAO overlayer thicknesses of 20 and 40 nm, as shown in Figure S5 in the Supporting Information. It is found that *I*_SD_–*V*_G_ curves show the hysteresis and irreversibility, especially for the first scan cycle, similar to that of the sample with a LAO thickness of 5 nm in Figure 1a. With the increasing of the LAO thickness, the *V*_G_ that is required to induce the insulating interface (low *I*_SD_) increases. The electrochemical oxygen migration is still active even for the LAO thickness of 40 nm, which also indicates that the oxygen ions in the amorphous LAO rather than that in the IL migrate into STO to fill in the vacancies during the gating. However, the upper limit of the thickness for the gating effect is not clear and would be a further study.

## 3 Conclusion

In conclusion, interface‐dependent electrochemical oxygen migration by IL gating is demonstrated in the LAO/STO 2DEG system. The correlation between interface structure and the mechanism of electrochemical oxygen migration are resolved through macroscopic transport measurements combined with atomic resolution STEM–EELS measurements. The interface structures, with different oxygen vacancy formation energies and energy barriers, lead to the filling of oxygen vacancies to different degrees, making the electrochemical oxygen migration a controllable process. Furthermore, the way to engineer the interface structure for electrochemical gating is discussed. Our results provide an atomic‐scale insight into interface‐dependent electrochemical gating, which can be generalized in the field of IL gating for a range of controllable devices.

## 4 Experimental Section

##### Electrical Gating Experiments

The LAO overlayers with a thickness of ≈5 nm were grown on (001) orientated STO substrates using a PLD system. The oxygen partial pressure *P*_O2_ was 2 × 10^−4 ^Torr for all samples. The Hall devices with a lateral gate electrode were fabricated as shown in Figure S6 in the Supporting Information. The width of the Hall bar was 200 µm and the distance between the two voltage probes was 160 µm. The patterning was done on STO before the deposition of the LAO overlayer by using conventional photolithography and the insulating amorphous AlN (*a*‐AlN) film was deposited as a hard mask. The gate electrode was formed by covering silver paint on the lateral gate pad. The wire connection for the transport measurement was done by Al ultrasonic wire bonding. During the gating experiment, a droplet of IL, *N*,*N*‐diethyl‐*N*‐methyl‐*N*‐(2‐methoxyethyl)ammonium bis(trifluoromethyl sulphonyl)imide (DEME‐TFSI), covered both the gate electrode and the Hall‐bar channel. To reduce the dissociation of water impurity in IL into proton and hydroxyl, the IL was dehydrated by keeping it in a vacuum (<10^−5 ^Torr) at 110 °C for more than 10 h before using it. The LAO/STO thin films were directly gated after the growth to avoid the exposure of the samples to chemical and water. Focused ion beam (FIB) was applied to specifically locate the gated areas of the Hall bar channel with an accuracy down to several nanometers and to further prepare the site‐specific specimens for STEM measurements using a standard lift‐out technique.

##### STEM–EELS Measurements

The cross‐sectional TEM specimens were prepared by focused ion beam milling with a Ga ion beam at 30 kV on an FEI Versa 3D machine, followed by thinning with low energy Ar^+^ in a Bal‐Tec Res‐120 ion beam milling system. The HAADF–STEM imaging and STEM–EELS experiments were carried out at 200 kV with an ARM200CF microscope equipped with a cold field‐emission electron gun, an ASCOR probe corrector, and Quantum dual‐EELS system. The collection angle for HAADF–STEM was 60–280 mrad and the convergence angle was 30 mrad. The EELS were acquired using a collection angle of 100 mrad with 0.03 s per pixel and a dispersion of 0.25 eV per channel. The elemental mapping was calculated based on the Digital Micrograph software.

## Conflict of Interest

The authors declare no conflict of interest.

## Supporting information

Supporting Information
