# Supplementary material for: Atomic Origin of Interface‐Dependent Oxygen Migration by Electrochemical Gating at the LaAlO3–SrTiO3 Heterointerface
Source: Adv Sci (Weinh). 2020 Jun 28;7(15):2000729. doi: 10.1002/advs.202000729 (PMC7404156; doi:10.1002/advs.202000729)
Supplement: Supplementary file 1 — Supporting Information [file ADVS-7-2000729-s001.pdf]

© 2020 Wiley-VCH GmbH

ADVANCED SCIENCE
Open Access

Supporting Information

for *Adv. Sci.*, DOI: 10.1002/advs.202000729

Atomic origin of interface-dependent oxygen migration by electrochemical gating at the $LaAlO_3$-$SrTiO_3$ heterointerface

Dongsheng Song, Deqing Xue, Shengwei Zeng, Changjian Li, Thirumalai Venkatesan, Ariando Ariando*, Stephen J. Pennycook**

Copyright WILEY-VCH Verlag GmbH & Co. KGaA, 69469 Weinheim, Germany, 2020.

Supporting Information

Atomic origin of interface-dependent oxygen migration by electrochemical gating at the $LaAlO_3$-$SrTiO_3$ heterointerface

Dongsheng Song, Deqing Xue, Shengwei Zeng[*]*, Changjian Li, Thirumalai Venkatesan, Ariando Ariando*[*]*, Stephen J. Pennycook*[*]

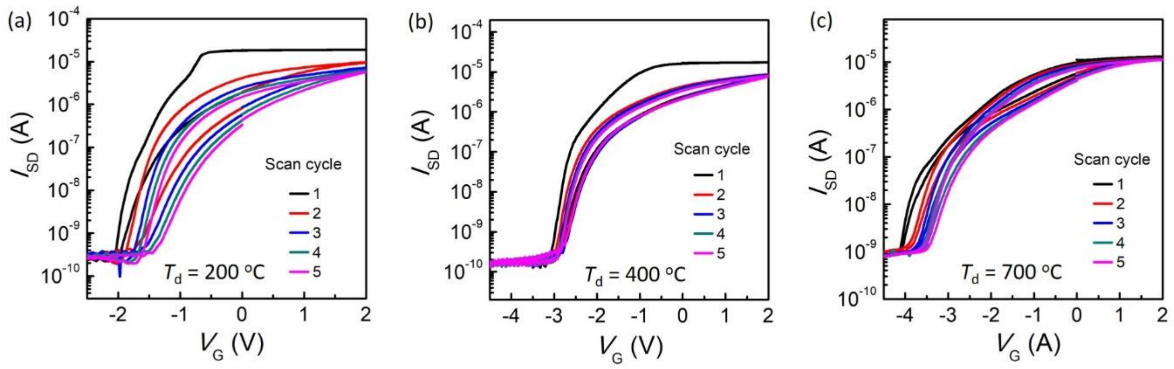

Figure S1. The source-drain current I_{SD} as a function of gate voltage V_G for the LAO/STO grown at T_d = 200, 400, and 700 ^oC, respectively.

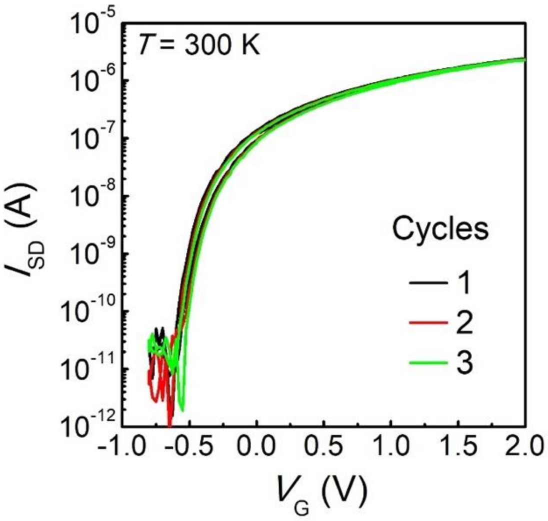

Figure S2. The source-drain current I_{SD} as a function of gate voltage V_G for the *c*-LAO/STO grown at 700 °C after oxygen annealing. The applied source-drain voltage is V_{SD} =3 V.

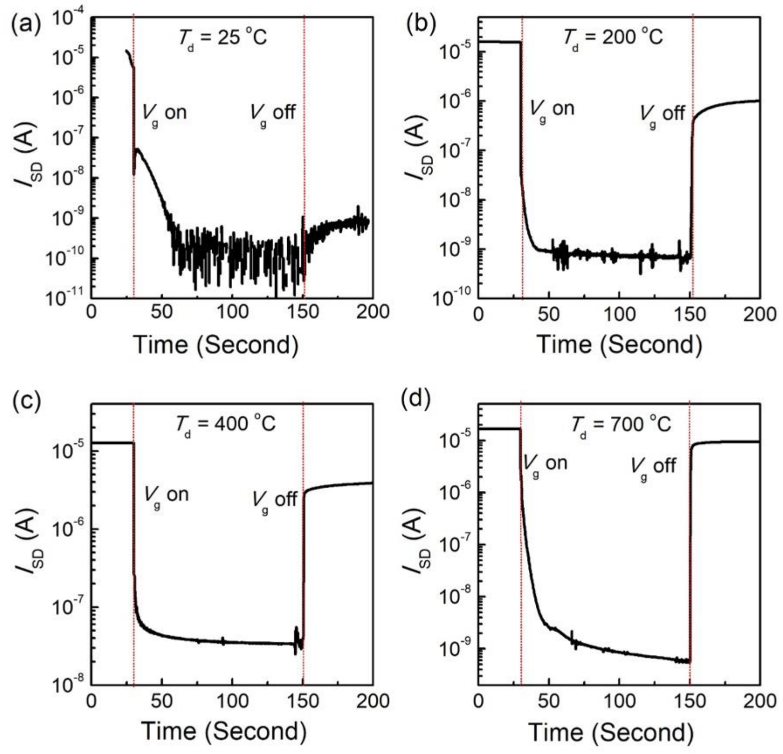

Figure S3. The source-drain current I_{SD} as a function of time as the negative gate voltage V_G is turned on for around 2 mins and then subsequently turned off for LAO/STO with deposition temperature T_d of 25, 200, 400 and 700 ^oC, respectively. For all of the samples, the V_G is -4 V and the source-drain voltage V_{SD} is 3 V.

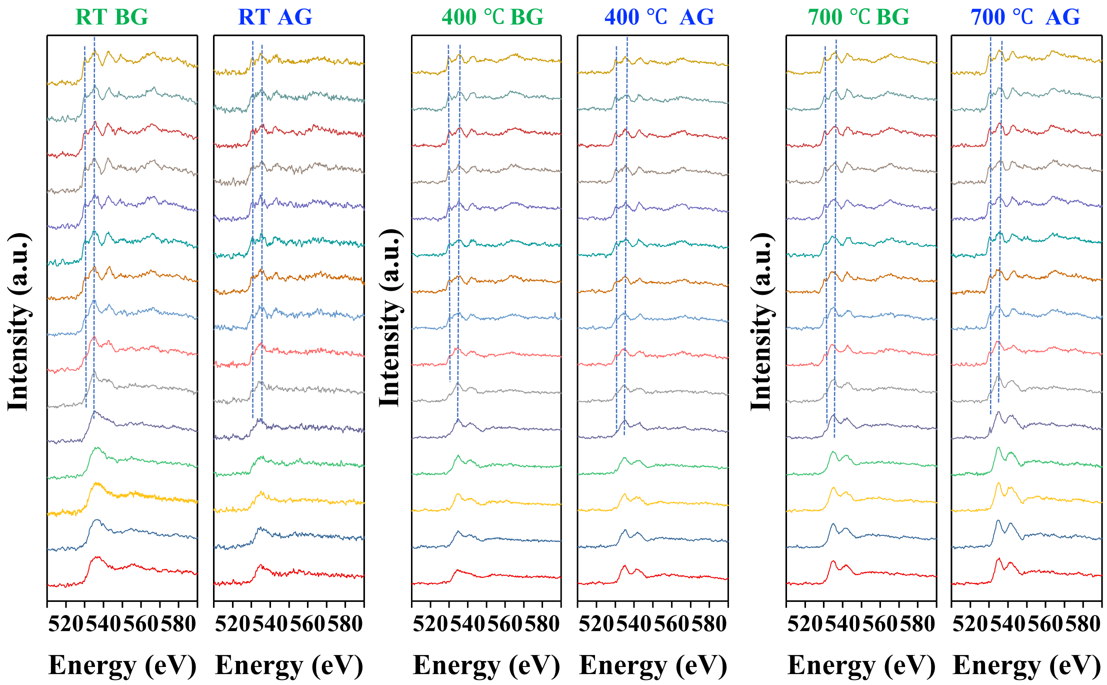

Figure S4. The EELS O *K* edge before and after IL gating across the LAO-STO interface for the samples grown at RT, 400 °C and 700 °C, respectively. The positions of peaks *a* and *b* are indicated with dotted lines.

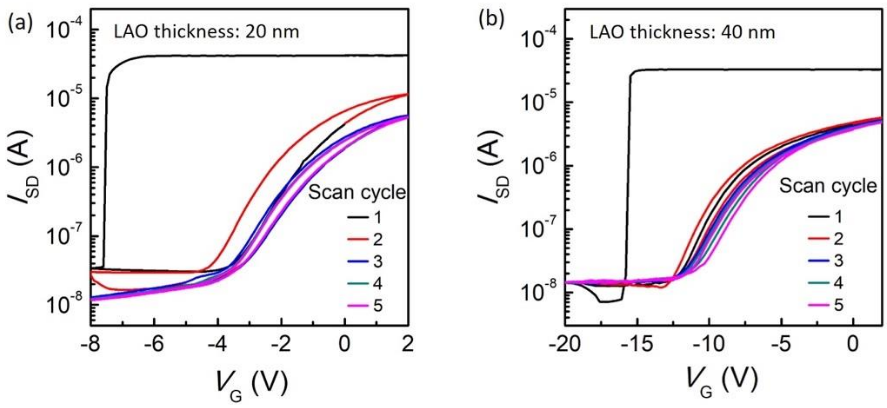

Figure S5. The source-drain current I_{SD} as a function of gate voltage V_G for the *a*-LAO/STO with LAO thicknesses of (a) 20 and (b) 40 nm at T_d = 25 ^oC. The applied source-drain voltage is V_{SD} =3 V.

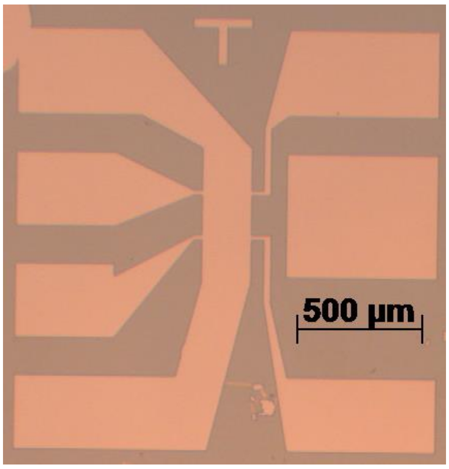

Figure S6. The Hall bar device pattern.
